# Exceptional serological and radiological response to sorafenib in 2 patients with advanced hepatocellular carcinoma and chronic hepatitis C viral infection: case report and review of the literature

**DOI:** 10.1186/s12876-017-0585-x

**Published:** 2017-02-14

**Authors:** Catherine Atkin, Philip Earwaker, Arvind Pallan, Shishir Shetty, Pankaj Punia, Yuk Ting Ma

**Affiliations:** 1 The Cancer Centre, University Hospitals Birmingham NHS Foundation Trust, Edgbaston, Birmingham, B15 2TH UK; 2 Department of Radiology, University Hospitals Birmingham NHS Foundation Trust, Edgbaston, Birmingham, B15 2TH UK; 3 The Liver Unit, University Hospitals Birmingham NHS Foundation Trust, Edgbaston, Birmingham, B15 2TH UK

**Keywords:** Sorafenib, Hepatocellular carcinoma, Hepatitis C virus infection

## Abstract

**Background:**

In patients with advanced hepatocellular carcinoma (HCC), the multikinase inhibitor sorafenib is the only systemic treatment that has been shown to increase overall survival. However, similar to other tyrosine kinase inhibitors, most patients achieve disease stabilisation radiologically, and only 2–3% of patients achieve a partial response. Recent exploratory subgroup analyses of the large phase 3 trials have demonstrated that patients with chronic hepatitis C virus (HCV) infection associated HCC survive longer than those who are negative for HCV. The mechanism underlying this currently remains unknown. A small number of cases of complete response to sorafenib treatment have now been reported worldwide, however a prolonged response has only been reported in 2 cases, both of whom had HCV-related HCC.

**Case presentation:**

A 55 year old gentleman was diagnosed with hepatocellular carcinoma and concomitant chronic hepatitis C viral infection. He progressed following transarterial chemoemoblisation treatment and was commenced on sorafenib treatment. His serum alphafetoprotein level normalised within 2 months of treatment and he achieved an almost complete radiological response. This response was maintained for 20 months before the patient progressed. A 75 year old lady was diagnosed with advanced hepatocellular carcinoma and concomitant chronic hepatitis C viral infection. She was commenced on sorafenib treatment but required early dose reductions due to palmar plantar erythrodysesthesia, and liver decompensation. Despite this she achieved an excellent serological and radiological response that was maintained for 24 months.

**Conclusions:**

Our two cases show that patients with HCV-associated HCC can attain excellent responses to sorafenib treatment that is durable. Furthermore, such exceptional responses can be achieved even with dose reductions and treatment breaks.

## Background

Hepatocellular carcinoma (HCC) is the 5th most common cancer worldwide, and the 3rd most common cause of cancer death. It often occurs on a background of chronic liver disease, including chronic hepatitis C (HCV) or hepatitis B (HBV) viral infection, and alcoholic liver disease [1].

Sorafenib, a multikinase inhibitor, is the only systemic treatment shown to increase overall survival in those with advanced HCC [2, 3]. However only 2–3% of patients achieve a partial response to sorafenib, with most patients achieving disease stabilisation.

Here we report two cases with advanced hepatocellular carcinoma and concomitant chronic hepatitis C viral infection, who achieved almost complete radiological responses to sorafenib treatment for a prolonged period. There are only 2 other reported cases in the literature reporting prolonged responses following sorafenib treatment [4, 5].

Chronic HCV infection is one of most common causes of HCC in Western countries. Despite the recent introduction of effective antiviral HCV therapy, the incidence of HCV-associated HCC is predicted to increase over the next few years due to the epidemic of undiagnosed chronic HCV infection [1]. There is now increasing evidence to suggest that patients with HCV-related HCC may attain superior survival benefit with sorafenib compared to patients who are negative for HCV. An exploratory subgroup analysis of the SHARP trial reported that patients with HCV-related HCC treated with sorafenib had a longer median overall survival compared to those with alcohol- or HBV-related HCC (14.0 vs 10.3 vs 9.7 months, respectively) [6]. In the phase III trial comparing sunitinib and sorafenib in patients with advanced HCC, median overall survival was superior in sorafenib treated patients, particularly in those with chronic HCV infection (17.6 vs 9.2 months) [7]. Similar results were was also reported in the phase III trial comparing brivanib and sorafenib; exploratory subgroup analyses demonstrated that the HCV positive subgroup of the sorafenib arm had a longer overall survival than the hepatitis C negative participants who received sorafenib (12.9 vs. 9.3 months) [8].

The precise mechanism underlying the possible higher efficacy of sorafenib in patients with chronic HCV infection currently remains unclear. In vitro, it has been shown that cellular expression of full length HCV enhances sensitivity to sorafenib. This was due to modulation of microRNA expression by the viral proteins, which in turn enhances apoptosis through downregulation of the anti-apoptotic protein Mcl-1 [9].

There is also increasing evidence outlining the mechanism by which sorafenib interacts with the hepatitis C virus. In vitro, sorafenib has been shown to inhibit HCV infection in a dose-dependent manner [10]. Multiple steps in the HCV infectious cycle appear to be affected, including inhibition of viral replication by blocking the c-Raf pathway that is normally activated by the virus, impaired secretion of HCV particles, and potent inhibition of viral entry and HCV cell-to-cell transmission [10, 11]. Further studies have researched whether sorafenib may have benefit in treating chronic HCV infection – however a small study of 33 patients being treated for HCC with sorafenib failed to show any significant change in measured viral load [12]. Similarly in our 2 cases, sorafenib treatment was not associated with any reduction in the HCV viral load.

## Case presentation 1

A 55 year old gentleman was diagnosed with hepatocellular carcinoma in September 2012, following investigations for deranged liver function tests. Magnetic resonance imaging (MRI) of the abdomen demonstrated a 4.5 cm liver lesion, with arterial phase hyperenhancement and venous phase washout, with an adjacent satellite nodule and invasion into a portal vein branch. The background liver appeared cirrhotic, and the spleen was enlarged. His serum alpha fetoprotein (AFP) was 1751kU/L, and a viral hepatitis screen showed chronic hepatitis C virus (HCV) infection and evidence of previous hepatitis B viral infection. His HCV viral load at baseline was 606926 IU/mL. He had well compensated liver function (Child Pugh class A) and an Eastern Cooperative Oncology Group (ECOG) performance status of zero.

**Fig. 1:**
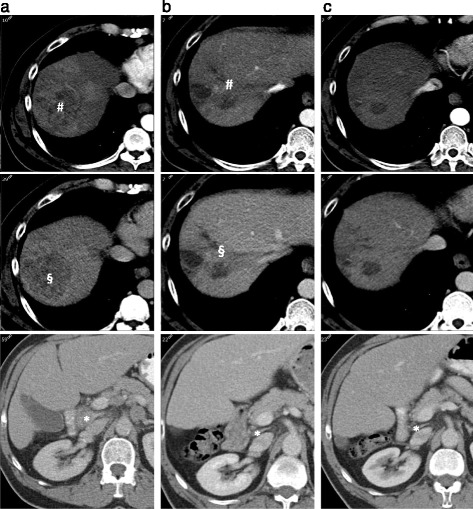
Case 1 response to sorafenib. **a**. Triple phase CT of the liver 07/12/2012. Primary HCC with arterial phase enhancement (# *top panel*) and portal venous washout (§ *middle panel*) with involved lymph node between portal vein and IVC (*bottom panel* *). **b**. Triple phase CT of the liver 03/04/2013. Primary HCC with reduced arterial phase enhancement (# *top panel*) and portal venous washout (§ *middle panel*) with two cystic areas. Reduction in size of lymph node (*bottom panel* *). **c**. Triple phase CT of the liver 15/07/2013. No arterial phase enhancement (*top panel*) or portal phase washout (*middle panel*) reflecting disappearance of tumour. Residual cystic areas. Sustained reduction in size of lymph node (*bottom panel* *)

He was treated with transarterial chemoembolisation (TACE) as the invasion into his branch portal vein was non-occlusive, but his computed tomography (CT) scan 4 weeks post-TACE demonstrated disease progression, with an increase in size and enhancement of the primary liver lesion, progression of the branch portal vein invasion and multiple new abdominal, para-aortic and mediastinal lymphadenopathy (Fig. 1a).

He started treatment with sorafenib in January 2013, achieving a maintenance dose of 600 mg daily after 2 months. He achieved an excellent serological response; his serum AFP level fell from a baseline value of 348kU/L to 5kU/L within 2 months and remained suppressed thereafter. This was associated with an excellent radiological response: CT imaging after 3 months of treatment showed a significant decrease in the size of the primary liver lesion and the lymphadenopathy (Fig. 1b). Follow-up CT imaging after 6 months of treatment demonstrated disappearance of all measurable disease apart from a residual lymph node adjacent to the caudate lobe (Fig. 1c). He maintained his excellent serological and radiological response for a further 14 months, until progressive disease was seen on repeat CT imaging in August 2014. He received a total of 20 months of treatment with sorafenib. Sorafenib had no effect on the patient’s HCV viral load, which remained significantly elevated during this period. The patient entered a second line clinical trial and remained alive for a further 11 months following discontinuation of sorafenib.

## Case presentation 2

**Fig. 2:**
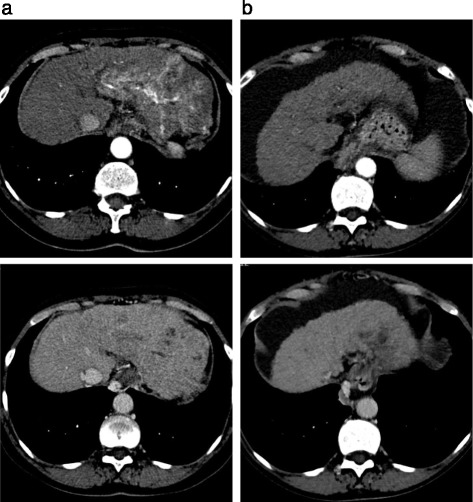
Case 2 response to sorafenib. **a**. Triple phase CT of the liver 10/06/2013. Primary HCC with arterial phase enhancement (*top panel*) and portal venous washout (*bottom panel*). **b**. Triple phase CT of the liver 19/12/2014. Primary HCC with no arterial phase enhancement (*top panel*) or portal venous washout (*bottom panel*)

A 75 year old lady was diagnosed with advanced hepatocellular carcinoma in June 2013 following investigations for low platelet count. A CT scan of the liver showed a 12 cm tumour in the left lobe with arterial phase hyperenhancement and venous phase washout, and left portal vein invasion (Fig. 2a). The background liver appeared cirrhotic. Her serum AFP level was 372 kU/L. A viral hepatitis screen confirmed chronic hepatitis C virus infection, with a low viral load (114 IU/mL). She had well compensated liver function (Child Pugh class A) and an ECOG performance status of 1.

She started sorafenib in July 2013, at a dose of 400 mg twice daily. After 7 days she developed grade 2 palmar-plantar erythrodysesthesia and treatment was paused and then restarted at reduced dose of 400 mg daily. She developed grade 2 hand-foot skin toxicity again and her sorafenib dose was thus reduced further to 200 mg daily, which was well tolerated. Repeat CT imaging after 3 months of treatment showed stable disease, however her serum AFP level had risen to 1574 kU/L. Her dose of sorafenib was cautiously increased. Over the next 2 months her serum AFP level declined rapidly to 6 kU/L.

Following this her liver synthetic function deteriorated and she decompensated with recurrent episodes of hepatic encephalopathy and ascites. Her treatment was paused for 4 months. Throughout this period her serum AFP level remained below 13kU/L. On review in March 2014 her liver synthetic function had improved (Child Pugh class B7), and she restarted low dose sorafenib. CT imaging in March showed ongoing stable disease despite the 4 month treatment break. Following resumption of sorafenib, serial CT scans showed reduction in the size of the liver lesion, with no tumour enhancement seen on her repeat imaging in December 2014 (Fig. 2b). She maintains her excellent serological and radiological response to date, 24 months after first starting sorafenib. Her HCV viral load initially increased to 4000 IU/mL after starting sorafenib and remains elevated.

## Conclusions

In patients with advanced HCC, sorafenib is the only systemic treatment that has been shown to increase overall survival [2, 3]. Sorafenib is an orally active, multikinase inhibitor that inhibits tumour angiogenesis and cell proliferation by blocking cell surface tyrosine kinases such as vascular endothelial growth factor receptor-2/3 (VEGFR-2/3) and platelet derived growth factor receptor beta, as well as downstream signalling pathways involving the serine/threonine kinases Raf-1 and B-Raf [13]. Its efficacy in treating HCC has been demonstrated in 2 randomised phase III trials [2, 3]. The SHARP trial, undertaken in patients with advanced HCC in Western countries, showed that patients with advanced hepatocellular carcinoma treated with sorafenib had a significant improvement in overall survival (10.7 months vs. 7.9 months, *p* < 0.001) and radiological time to progression (5.5 months vs. 2.8 months, *p* < 0.001) when compared to placebo [2]. The Asia-Pacific trial also showed that sorafenib significantly prolonged overall survival (6.5 months vs. 4.2 months, hazard ratio 0.68, *p* = 0.014) compared to placebo, in patients from the Asia-Pacific region [3]. In both trials a partial response was observed in only a small minority of patients (2 and 3% respectively) and no complete responses were seen.

A small number of cases of complete response to sorafenib treatment have now been reported worldwide [4, 5, 14–17], however a prolonged response (>18 months) has only been reported in 2 cases, both of whom had HCV-related HCC [4, 5]. Both of our cases also had HCV-related HCC and demonstrated prolonged responses (20 and 24 months, respectively) to sorafenib treatment. The 2 cases presented here add to the accumulating evidence that patients with HCV-related HCC may attain superior survival benefit with sorafenib compared to patients who are negative for HCV. The second case is particularly noteworthy, because the prolonged response was achieved even with dose reductions and a treatment break (due to decompensation), and highlights that even with dose reductions, significant response to sorafenib can be achieved.

## Abbreviations

AFP: Alpha fetoprotein
CT: Computed tomography
ECOG: Eastern cooperative oncology group
HBV: Hepatitis B virus infection
HCC: Hepatocellular carcinoma
HCV: Hepatitis C virus infection
MRI: Magnetic resonance imaging
RNA: Ribonucleic acid
TACE: Transarterial chemoembolization
VEGFR: Vascular endothelial growth factor receptor
